# Optimizing citrate dose for regional anticoagulation in continuous renal replacement therapy: measuring citrate concentrations instead of ionized calcium?

**DOI:** 10.1186/s13054-015-1103-6

**Published:** 2015-11-06

**Authors:** Patrick M. Honore, Rita Jacobs, Inne Hendrickx, Elisabeth De Waele, Viola Van Gorp, Herbert D. Spapen

**Affiliations:** ICU Department, Universitair Ziekenhuis Brussel, Vrije Universiteit Brussel, 101, Laarbeeklaan, 1090 Jette Brussels, Belgium

Regular measurement of systemic and post-filter ionized calcium (iCa) concentrations is imperative to correctly handle regional citrate anticoagulation dose during continuous renal replacement therapy (CRRT). Keeping post-filter iCa within a tight range guarantees optimal circuit function and enhances filter life span [1, 2], whereas a decrease in plasma iCa, with subsequent elevation of the total-to-ionized plasma calcium ratio, can predict systemic citrate accumulation [3].

The new findings (published recently in *Critical Care*) of Schwarzer et al. expose an alarming inaccuracy for measuring post-filter iCa with currently available blood gas analyzers [4]. This precludes adequate control of citrate flow and raises evident functional and safety issues. On the other hand, Schwarzer et al. found good concordance between all evaluated analyzers for measuring systemic iCa levels [4]. However, the total-to-ionized plasma calcium ratio has occasionally been shown to be a relatively weak indirect marker for citrate accumulation or intoxication [1, 2]. Direct measurement of citrate systemic concentrations could overcome these iCa-related shortcomings. In this perspective, compelling evidence was provided by Italian investigators who adapted a commercially available citrate analyzing kit for measuring systemic and also filter citrate concentrations [5]. Preliminary experience in septic shock patients with liver dysfunction undergoing CRRT suggested a potential clinical use but needs confirmation in a larger and more heterogeneous patient population.

## Abbreviations

CRRT: Continuous renal replacement therapy
iCa: Ionized calcium
